# 4′-(3-Bromo­phen­yl)-1′-methyl­dispiro­[indan-2,2′-pyrrolidine-3′,2′′-indan]-1,3,1′′-trione

**DOI:** 10.1107/S1600536812037993

**Published:** 2012-09-08

**Authors:** Ang Chee Wei, Mohamed Ashraf Ali, Tan Soo Choon, Suhana Arshad, Ibrahim Abdul Razak

**Affiliations:** a Institute for Research in Molecular Medicine, Universiti Sains Malaysia, Minden 11800, Penang, Malaysia; b School of Physics, Universiti Sains Malaysia, 11800 USM, Penang, Malaysia

## Abstract

In the title compound, C_27_H_20_BrNO_3_, two intra­molecular C—H⋯O hydrogen bonds both form *S*(6) rings. The pyrrolidine ring adopts a twisted conformation about the C—C bond bearing the indane ring systems. The other two five-membered rings within the indane systems are in shallow envelope conformations, with the spiro C atoms as the flap atoms. The mean plane of the pyrrolidine ring [maximum deviation = 0.275 (1) Å] makes dihedral angles of 65.25 (7), 78.33 (6) and 75.25 (6)° with the bromo-substituted benzene ring and the mean planes of the mono- and dioxo-substituted indane rings, respectively. In the crystal, mol­ecules are linked by C—H⋯O and C—H⋯N hydrogen bonds into a three-dimensional network. In addition, C—H⋯π inter­actions are observed.

## Related literature

For related structures and medicinal background, see: Wei *et al.* (2011 ▶, 2012*a* ▶,*b* ▶). For ring conformations, see: Cremer & Pople (1975 ▶). For hydrogen-bond motifs, see: Bernstein *et al.* (1995 ▶). For the stability of the temperature controller used in the data collection, see: Cosier & Glazer (1986 ▶).
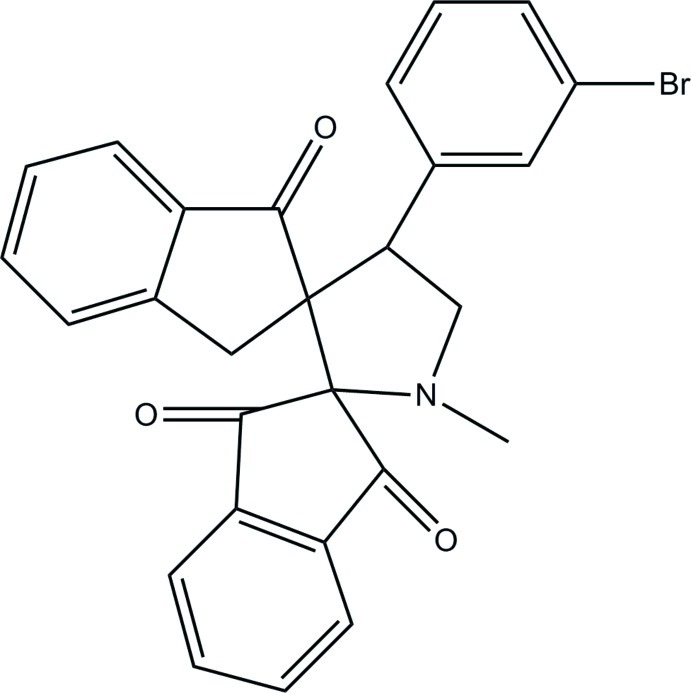


## Experimental

### 

#### Crystal data

C_27_H_20_BrNO_3_
*M* *_r_* = 486.35
Triclinic, 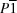

*a* = 8.3998 (1) Å
*b* = 11.2082 (2) Å
*c* = 12.4816 (2) Å
α = 112.004 (1)°
β = 96.850 (1)°
γ = 93.191 (1)°
*V* = 1075.28 (3) Å^3^
*Z* = 2
Mo *K*α radiation
μ = 1.94 mm^−1^
*T* = 100 K
0.51 × 0.31 × 0.29 mm

#### Data collection

Bruker SMART APEXII CCD diffractometer
Absorption correction: multi-scan (*SADABS*; Bruker, 2009 ▶) *T*_min_ = 0.436, *T*_max_ = 0.599
23814 measured reflections
6318 independent reflections
5810 reflections with *I* > 2σ(*I*)
*R*_int_ = 0.023

#### Refinement

*R*[*F* ^2^ > 2σ(*F* ^2^)] = 0.028
*wR*(*F* ^2^) = 0.075
*S* = 1.04
6318 reflections
290 parameters
H-atom parameters constrained
Δρ_max_ = 0.52 e Å^−3^
Δρ_min_ = −0.53 e Å^−3^

### 

Data collection: *APEX2* (Bruker, 2009 ▶); cell refinement: *SAINT* (Bruker, 2009 ▶); data reduction: *SAINT*; program(s) used to solve structure: *SHELXTL* (Sheldrick, 2008 ▶); program(s) used to refine structure: *SHELXTL*; molecular graphics: *SHELXTL*; software used to prepare material for publication: *SHELXTL* and *PLATON* (Spek, 2009 ▶).

## Supplementary Material

Crystal structure: contains datablock(s) global, I. DOI: 10.1107/S1600536812037993/hb6949sup1.cif

Structure factors: contains datablock(s) I. DOI: 10.1107/S1600536812037993/hb6949Isup2.hkl

Additional supplementary materials:  crystallographic information; 3D view; checkCIF report

## Figures and Tables

**Table 1:** Hydrogen-bond geometry (Å, °) *Cg*1 is the centroid of the C21–C26 ring.

*D*—H⋯*A*	*D*—H	H⋯*A*	*D*⋯*A*	*D*—H⋯*A*
C18—H18*B*⋯O2	0.99	2.39	3.0787 (17)	126
C19—H19*A*⋯O1	1.00	2.53	3.1649 (17)	121
C4—H4*A*⋯O3^i^	0.95	2.53	3.4056 (18)	153
C16—H16*A*⋯N1^ii^	0.95	2.58	3.4912 (16)	161
C20—H20*B*⋯O1^iii^	0.99	2.44	3.3586 (18)	153
C23—H23*A*⋯O1^iv^	0.95	2.48	3.3847 (18)	160
C5—H5*A*⋯*Cg*1^v^	0.95	2.65	3.3386 (17)	130
C15—H15*A*⋯*Cg*1^vi^	0.95	2.82	3.6623 (15)	149

Symmetry codes: (i) 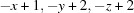; (ii) 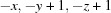; (iii) 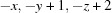; (iv) 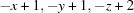; (v) 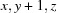; (vi) 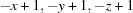.

**Fig. 1.**
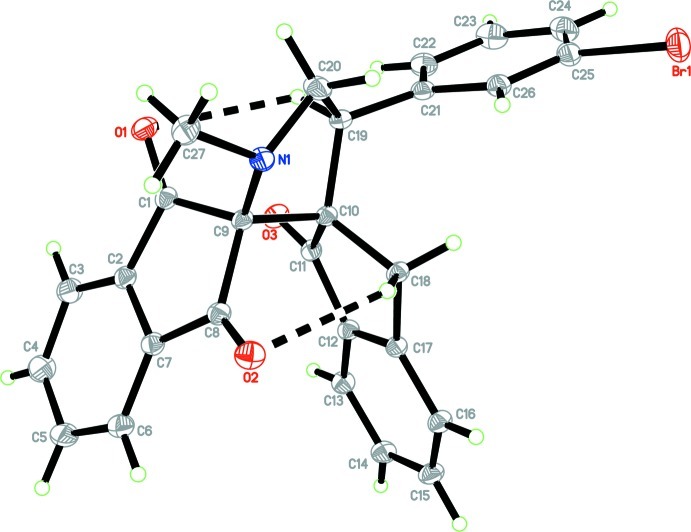
The molecular structure of the title compound, showing 50% probability displacement ellipsoids.

**Fig. 2.**
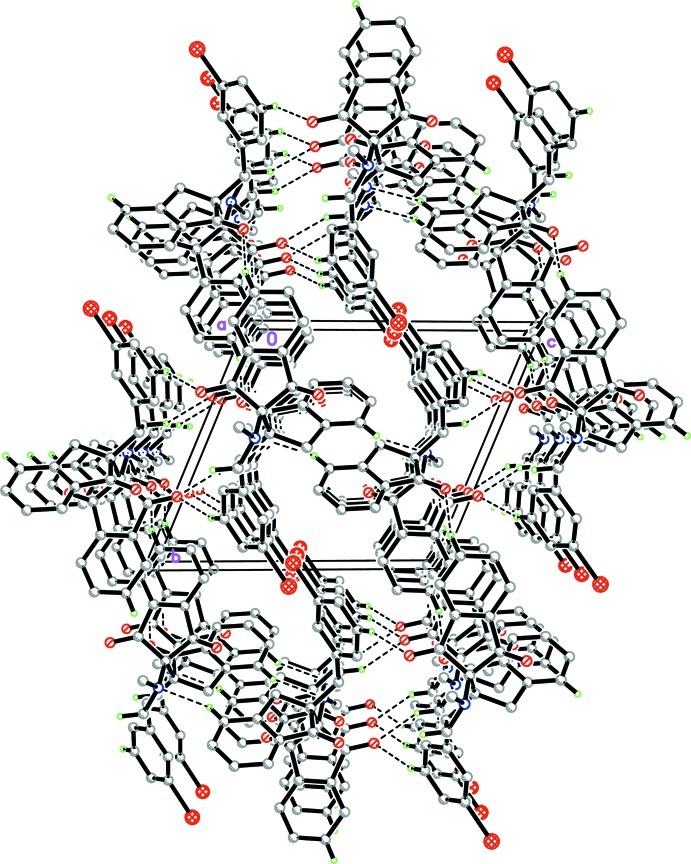
The crystal packing of the title compound. The H atoms not involved in the intermolecular interactions (dashed lines) have been omitted for clarity.

## supplementary crystallographic information

### Comment

Tuberculosis (TB) remains a global health problem and has infected about one third of the world population. No new drugs have been discovered for the past 40 years and therefore new anti-TB agents are desperately needed. As part of our ongoing search for novel heterocyclic compounds with antitubercular activity (Wei *et al.*, 2011;, 2012*a*,*b*), our group has synthesized the title compound as described below.

The molecular structure of the title compound is shown in Fig. 1. The intramolecular C18—H18B···O2 and C19—H19A···O1 hydrogen bonds (Table 1) form two *S*(6) ring motifs (Bernstein *et al.*, 1995). The pyrrolidine ring (N1/C9/C10/C19/C20) is twisted about C9–C10 bond [puckering parameters, Q= 0.4492 (14) Å and φ= 242.64 (18)°], thereby adopting half-chair comformation (Cremer & Pople, 1975). Meanwhile, the other two five-membered rings within the indane moiety (C1/C2/C7–C9 & C10–C12/C17/C18) are in envelope conformation with puckering parameters, Q = 0.1825 (14) Å and φ= 148.5 (4)° in which C9 at the flap and Q = 0.1694 (14) Å and φ= 172.8 (5)° in which C10 at the flap, respectively. The mean plane of the pyrrolidine ring [N1/C9/C10/C19/C20, with maximum deviation of 0.275 (1) Å at atom C10] makes dihedral angles of 65.25 (7), 78.33 (6) and 75.25 (6)° with the bromo-substituted benzene ring (C21–C26) and the mean planes of the mono-oxo substituted [C10–C18; maximum deviation of 0.145 (1) Å at atom C10] and di-oxo substituted [C1–C9; maximum deviation of 0.180 (1) Å at atom C9] indane rings, respectively. The bond lengths and angles are within normal ranges and comparable to the related structure (Wei, Ali, Yoon *et al.*, 2011; Wei, Ali, Choon *et al.*, 2012*a*,*b*).

In the crystal (Fig. 2), C4—H4A···O3, C20—H20B···O1, C16—H16A···N1 and C23—H23A···O1 hydrogen bonds (Table 1) connect the molecules into a three-dimensional network. The crystal structure are further stabilized by the intermolecular C5—H5A···*Cg*1 and C15—H15A···*Cg*1 (Table 1) interactions (*Cg*1 is the centroid of C21–C26).

### Experimental

A mixture of (*E*)-2-(3-bromobenzylidene)-2,3-dihydro-1*H*-indene-1 -one (0.001 mol), ninhydrin (0.001 mol) and sarcosine (0.002 mol) (1:1:2) were dissolved in methanol (10 ml) and refluxed for 4 h. After completion of the reaction as evident from TLC, the excess solvent was evaporated slowly and the product was separated and recrystallized from methanol to reveal the title compound as yellow blocks.

### Refinement

All H atoms were positioned geometrically [C–H = 0.95 and 1.00 Å] and refined using a riding model with *U*_iso_(H) = 1.2 and 1.5 *U*_eq_(C). A rotating group model was applied to the methyl group. One outliner -9 -1 2 was omitted in the final refinement.

### Crystal data

**Table:**

C_27_H_20_BrNO_3_	*Z* = 2
*M**_r_* = 486.35	*F*(000) = 496
Triclinic, *P*1	*D*_x_ = 1.502 Mg m^−^^3^
Hall symbol: -P 1	Mo *K*α radiation, λ = 0.71073 Å
*a* = 8.3998 (1) Å	Cell parameters from 9917 reflections
*b* = 11.2082 (2) Å	θ = 3.0–30.1°
*c* = 12.4816 (2) Å	µ = 1.94 mm^−^^1^
α = 112.004 (1)°	*T* = 100 K
β = 96.850 (1)°	Block, yellow
γ = 93.191 (1)°	0.51 × 0.31 × 0.29 mm
*V* = 1075.28 (3) Å^3^	

### Data collection

**Table:**

Bruker SMART APEXII CCD diffractometer	6318 independent reflections
Radiation source: fine-focus sealed tube	5810 reflections with *I* > 2σ(*I*)
Graphite monochromator	*R*_int_ = 0.023
φ and ω scans	θ_max_ = 30.1°, θ_min_ = 1.8°
Absorption correction: multi-scan (*SADABS*; Bruker, 2009)	*h* = −11→11
*T*_min_ = 0.436, *T*_max_ = 0.599	*k* = −15→15
23814 measured reflections	*l* = −17→17

### Refinement

**Table:**

Refinement on *F*^2^	Primary atom site location: structure-invariant direct methods
Least-squares matrix: full	Secondary atom site location: difference Fourier map
*R*[*F*^2^ > 2σ(*F*^2^)] = 0.028	Hydrogen site location: inferred from neighbouring sites
*wR*(*F*^2^) = 0.075	H-atom parameters constrained
*S* = 1.04	*w* = 1/[σ^2^(*F*_o_^2^) + (0.0394*P*)^2^ + 0.5548*P*] where *P* = (*F*_o_^2^ + 2*F*_c_^2^)/3
6318 reflections	(Δ/σ)_max_ = 0.001
290 parameters	Δρ_max_ = 0.52 e Å^−^^3^
0 restraints	Δρ_min_ = −0.53 e Å^−^^3^

### Special details

**Table:**

Experimental. The crystal was placed in the cold stream of an Oxford Cryosystems Cobra open-flow nitrogen cryostat (Cosier & Glazer, 1986) operating at 100.0 (1) K.
Geometry. All e.s.d.'s (except the e.s.d. in the dihedral angle between two l.s. planes) are estimated using the full covariance matrix. The cell e.s.d.'s are taken into account individually in the estimation of e.s.d.'s in distances, angles and torsion angles; correlations between e.s.d.'s in cell parameters are only used when they are defined by crystal symmetry. An approximate (isotropic) treatment of cell e.s.d.'s is used for estimating e.s.d.'s involving l.s. planes.
Refinement. Refinement of *F*^2^ against ALL reflections. The weighted *R*-factor *wR* and goodness of fit *S* are based on *F*^2^, conventional *R*-factors *R* are based on *F*, with *F* set to zero for negative *F*^2^. The threshold expression of *F*^2^ > σ(*F*^2^) is used only for calculating *R*-factors(gt) *etc*. and is not relevant to the choice of reflections for refinement. *R*-factors based on *F*^2^ are statistically about twice as large as those based on *F*, and *R*- factors based on ALL data will be even larger.

### Fractional atomic coordinates and isotropic or equivalent isotropic displacement parameters (Å^2^)

**Table:**

	*x*	*y*	*z*	*U*_iso_*/*U*_eq_	
Br1	0.225327 (19)	−0.034338 (13)	0.491671 (13)	0.02629 (5)	
O1	0.13721 (12)	0.70139 (9)	1.01601 (8)	0.01768 (18)	
O2	−0.06941 (12)	0.68997 (9)	0.64765 (8)	0.01841 (18)	
O3	0.45101 (11)	0.66808 (9)	0.88616 (8)	0.01675 (18)	
N1	−0.05884 (13)	0.52853 (10)	0.78612 (9)	0.01380 (19)	
C1	0.12730 (14)	0.72671 (12)	0.92921 (10)	0.0133 (2)	
C2	0.15991 (15)	0.85606 (12)	0.92485 (11)	0.0147 (2)	
C3	0.24222 (17)	0.96921 (13)	1.01069 (12)	0.0197 (3)	
H3A	0.2879	0.9714	1.0849	0.024*	
C4	0.25502 (19)	1.07871 (13)	0.98385 (12)	0.0233 (3)	
H4A	0.3133	1.1565	1.0398	0.028*	
C5	0.18338 (19)	1.07629 (13)	0.87559 (13)	0.0226 (3)	
H5A	0.1907	1.1534	0.8606	0.027*	
C6	0.10196 (17)	0.96343 (13)	0.78980 (12)	0.0188 (2)	
H6A	0.0534	0.9617	0.7164	0.023*	
C7	0.09426 (15)	0.85257 (12)	0.81561 (11)	0.0144 (2)	
C8	0.01860 (14)	0.72063 (12)	0.73996 (11)	0.0134 (2)	
C9	0.06749 (14)	0.62884 (11)	0.80214 (10)	0.0118 (2)	
C10	0.20794 (14)	0.54710 (11)	0.75111 (10)	0.0117 (2)	
C11	0.37225 (14)	0.63118 (11)	0.78907 (11)	0.0128 (2)	
C12	0.41207 (14)	0.65657 (12)	0.68743 (10)	0.0128 (2)	
C13	0.53931 (15)	0.73898 (12)	0.68183 (11)	0.0152 (2)	
H13A	0.6138	0.7891	0.7497	0.018*	
C14	0.55375 (16)	0.74547 (13)	0.57472 (12)	0.0172 (2)	
H14A	0.6395	0.8002	0.5683	0.021*	
C15	0.44190 (16)	0.67141 (13)	0.47562 (11)	0.0172 (2)	
H15A	0.4525	0.6778	0.4030	0.021*	
C16	0.31575 (15)	0.58883 (12)	0.48106 (11)	0.0152 (2)	
H16A	0.2412	0.5388	0.4132	0.018*	
C17	0.30179 (14)	0.58155 (11)	0.58869 (10)	0.0126 (2)	
C18	0.18261 (15)	0.49507 (12)	0.61636 (10)	0.0129 (2)	
H18A	0.2052	0.4037	0.5829	0.016*	
H18B	0.0707	0.5013	0.5852	0.016*	
C19	0.19356 (15)	0.44614 (12)	0.80828 (11)	0.0137 (2)	
H19A	0.2382	0.4924	0.8930	0.016*	
C20	0.01072 (16)	0.41788 (13)	0.80165 (13)	0.0186 (2)	
H20A	−0.0317	0.3363	0.7348	0.022*	
H20B	−0.0147	0.4104	0.8745	0.022*	
C21	0.28456 (15)	0.32899 (12)	0.76277 (11)	0.0141 (2)	
C22	0.43223 (16)	0.32640 (13)	0.82660 (12)	0.0183 (2)	
H22A	0.4753	0.3995	0.8952	0.022*	
C23	0.51708 (17)	0.21825 (15)	0.79110 (14)	0.0230 (3)	
H23A	0.6175	0.2186	0.8353	0.028*	
C24	0.45598 (17)	0.11009 (14)	0.69174 (14)	0.0224 (3)	
H24A	0.5124	0.0356	0.6678	0.027*	
C25	0.31020 (17)	0.11346 (12)	0.62810 (12)	0.0182 (2)	
C26	0.22394 (15)	0.22029 (12)	0.66123 (11)	0.0156 (2)	
H26A	0.1248	0.2198	0.6156	0.019*	
C27	−0.19926 (16)	0.56775 (13)	0.84332 (12)	0.0185 (2)	
H27A	−0.2416	0.6379	0.8236	0.028*	
H27B	−0.1682	0.5980	0.9282	0.028*	
H27C	−0.2825	0.4938	0.8167	0.028*	

### Atomic displacement parameters (Å^2^)

**Table:**

	*U*^11^	*U*^22^	*U*^33^	*U*^12^	*U*^13^	*U*^23^
Br1	0.03101 (9)	0.01684 (7)	0.02711 (8)	0.00156 (5)	0.01300 (6)	0.00162 (5)
O1	0.0203 (4)	0.0199 (4)	0.0145 (4)	0.0010 (4)	0.0044 (3)	0.0082 (4)
O2	0.0171 (4)	0.0200 (4)	0.0177 (4)	0.0007 (3)	−0.0005 (3)	0.0080 (4)
O3	0.0142 (4)	0.0212 (4)	0.0140 (4)	−0.0012 (3)	0.0008 (3)	0.0067 (3)
N1	0.0116 (4)	0.0132 (4)	0.0174 (5)	−0.0003 (4)	0.0051 (4)	0.0062 (4)
C1	0.0120 (5)	0.0145 (5)	0.0135 (5)	0.0006 (4)	0.0039 (4)	0.0052 (4)
C2	0.0161 (5)	0.0141 (5)	0.0140 (5)	0.0001 (4)	0.0048 (4)	0.0051 (4)
C3	0.0250 (7)	0.0161 (6)	0.0157 (6)	−0.0022 (5)	0.0023 (5)	0.0045 (5)
C4	0.0316 (7)	0.0152 (6)	0.0200 (6)	−0.0049 (5)	0.0047 (5)	0.0041 (5)
C5	0.0326 (7)	0.0151 (6)	0.0222 (6)	−0.0016 (5)	0.0081 (5)	0.0088 (5)
C6	0.0236 (6)	0.0172 (6)	0.0179 (6)	0.0008 (5)	0.0059 (5)	0.0088 (5)
C7	0.0145 (5)	0.0141 (5)	0.0150 (5)	0.0004 (4)	0.0044 (4)	0.0057 (4)
C8	0.0121 (5)	0.0149 (5)	0.0148 (5)	0.0016 (4)	0.0044 (4)	0.0069 (4)
C9	0.0119 (5)	0.0124 (5)	0.0117 (5)	−0.0003 (4)	0.0030 (4)	0.0051 (4)
C10	0.0110 (5)	0.0126 (5)	0.0118 (5)	−0.0006 (4)	0.0029 (4)	0.0050 (4)
C11	0.0115 (5)	0.0129 (5)	0.0143 (5)	0.0011 (4)	0.0039 (4)	0.0050 (4)
C12	0.0120 (5)	0.0141 (5)	0.0135 (5)	0.0014 (4)	0.0034 (4)	0.0062 (4)
C13	0.0130 (5)	0.0160 (5)	0.0168 (5)	−0.0006 (4)	0.0026 (4)	0.0066 (4)
C14	0.0160 (6)	0.0177 (5)	0.0203 (6)	−0.0007 (4)	0.0060 (5)	0.0094 (5)
C15	0.0186 (6)	0.0201 (6)	0.0166 (5)	0.0023 (5)	0.0065 (4)	0.0102 (5)
C16	0.0154 (5)	0.0169 (5)	0.0138 (5)	0.0003 (4)	0.0035 (4)	0.0063 (4)
C17	0.0125 (5)	0.0129 (5)	0.0137 (5)	0.0014 (4)	0.0042 (4)	0.0058 (4)
C18	0.0133 (5)	0.0140 (5)	0.0112 (5)	−0.0018 (4)	0.0026 (4)	0.0048 (4)
C19	0.0157 (5)	0.0136 (5)	0.0140 (5)	0.0011 (4)	0.0048 (4)	0.0069 (4)
C20	0.0171 (6)	0.0155 (5)	0.0280 (7)	0.0027 (4)	0.0109 (5)	0.0114 (5)
C21	0.0150 (5)	0.0147 (5)	0.0157 (5)	0.0014 (4)	0.0053 (4)	0.0084 (4)
C22	0.0161 (6)	0.0201 (6)	0.0205 (6)	0.0007 (5)	0.0022 (5)	0.0101 (5)
C23	0.0160 (6)	0.0273 (7)	0.0309 (7)	0.0049 (5)	0.0034 (5)	0.0167 (6)
C24	0.0211 (6)	0.0215 (6)	0.0313 (7)	0.0083 (5)	0.0116 (5)	0.0148 (6)
C25	0.0204 (6)	0.0148 (5)	0.0208 (6)	0.0015 (5)	0.0092 (5)	0.0067 (5)
C26	0.0163 (5)	0.0160 (5)	0.0168 (5)	0.0020 (4)	0.0052 (4)	0.0081 (5)
C27	0.0138 (5)	0.0205 (6)	0.0226 (6)	0.0018 (4)	0.0074 (5)	0.0084 (5)

### Geometric parameters (Å, º)

**Table:**

Br1—C25	1.9019 (14)	C13—H13A	0.9500
O1—C1	1.2135 (15)	C14—C15	1.4030 (18)
O2—C8	1.2100 (15)	C14—H14A	0.9500
O3—C11	1.2165 (15)	C15—C16	1.3925 (17)
N1—C9	1.4416 (15)	C15—H15A	0.9500
N1—C27	1.4553 (16)	C16—C17	1.3943 (16)
N1—C20	1.4665 (16)	C16—H16A	0.9500
C1—C2	1.4827 (17)	C17—C18	1.5120 (16)
C1—C9	1.5544 (17)	C18—H18A	0.9900
C2—C3	1.3935 (17)	C18—H18B	0.9900
C2—C7	1.3939 (17)	C19—C21	1.5123 (17)
C3—C4	1.3900 (19)	C19—C20	1.5369 (18)
C3—H3A	0.9500	C19—H19A	1.0000
C4—C5	1.402 (2)	C20—H20A	0.9900
C4—H4A	0.9500	C20—H20B	0.9900
C5—C6	1.3887 (19)	C21—C22	1.4000 (18)
C5—H5A	0.9500	C21—C26	1.4035 (18)
C6—C7	1.3956 (17)	C22—C23	1.3938 (19)
C6—H6A	0.9500	C22—H22A	0.9500
C7—C8	1.4793 (17)	C23—C24	1.387 (2)
C8—C9	1.5488 (16)	C23—H23A	0.9500
C9—C10	1.5809 (17)	C24—C25	1.388 (2)
C10—C18	1.5427 (16)	C24—H24A	0.9500
C10—C11	1.5434 (16)	C25—C26	1.3881 (18)
C10—C19	1.5540 (16)	C26—H26A	0.9500
C11—C12	1.4708 (16)	C27—H27A	0.9800
C12—C17	1.3959 (17)	C27—H27B	0.9800
C12—C13	1.3988 (16)	C27—H27C	0.9800
C13—C14	1.3849 (17)		
			
C9—N1—C27	117.31 (10)	C16—C15—C14	121.67 (11)
C9—N1—C20	110.09 (10)	C16—C15—H15A	119.2
C27—N1—C20	115.37 (10)	C14—C15—H15A	119.2
O1—C1—C2	126.85 (11)	C15—C16—C17	118.25 (11)
O1—C1—C9	125.46 (11)	C15—C16—H16A	120.9
C2—C1—C9	107.59 (10)	C17—C16—H16A	120.9
C3—C2—C7	121.15 (11)	C16—C17—C12	119.94 (11)
C3—C2—C1	129.27 (11)	C16—C17—C18	128.53 (11)
C7—C2—C1	109.57 (11)	C12—C17—C18	111.46 (10)
C4—C3—C2	117.60 (13)	C17—C18—C10	104.24 (9)
C4—C3—H3A	121.2	C17—C18—H18A	110.9
C2—C3—H3A	121.2	C10—C18—H18A	110.9
C3—C4—C5	121.12 (13)	C17—C18—H18B	110.9
C3—C4—H4A	119.4	C10—C18—H18B	110.9
C5—C4—H4A	119.4	H18A—C18—H18B	108.9
C6—C5—C4	121.28 (12)	C21—C19—C20	115.88 (10)
C6—C5—H5A	119.4	C21—C19—C10	116.93 (10)
C4—C5—H5A	119.4	C20—C19—C10	103.69 (10)
C5—C6—C7	117.38 (12)	C21—C19—H19A	106.5
C5—C6—H6A	121.3	C20—C19—H19A	106.5
C7—C6—H6A	121.3	C10—C19—H19A	106.5
C2—C7—C6	121.37 (12)	N1—C20—C19	105.19 (10)
C2—C7—C8	110.26 (10)	N1—C20—H20A	110.7
C6—C7—C8	128.36 (11)	C19—C20—H20A	110.7
O2—C8—C7	126.41 (11)	N1—C20—H20B	110.7
O2—C8—C9	125.88 (11)	C19—C20—H20B	110.7
C7—C8—C9	107.69 (10)	H20A—C20—H20B	108.8
N1—C9—C8	113.45 (10)	C22—C21—C26	118.48 (12)
N1—C9—C1	117.20 (10)	C22—C21—C19	118.89 (11)
C8—C9—C1	101.52 (9)	C26—C21—C19	122.59 (11)
N1—C9—C10	101.75 (9)	C23—C22—C21	121.04 (13)
C8—C9—C10	113.18 (9)	C23—C22—H22A	119.5
C1—C9—C10	110.18 (9)	C21—C22—H22A	119.5
C18—C10—C11	104.54 (9)	C24—C23—C22	120.48 (13)
C18—C10—C19	117.18 (10)	C24—C23—H23A	119.8
C11—C10—C19	113.68 (10)	C22—C23—H23A	119.8
C18—C10—C9	111.30 (9)	C23—C24—C25	118.31 (13)
C11—C10—C9	111.58 (9)	C23—C24—H24A	120.8
C19—C10—C9	98.74 (9)	C25—C24—H24A	120.8
O3—C11—C12	127.83 (11)	C26—C25—C24	122.29 (13)
O3—C11—C10	124.83 (11)	C26—C25—Br1	119.05 (10)
C12—C11—C10	107.34 (10)	C24—C25—Br1	118.66 (10)
C17—C12—C13	121.81 (11)	C25—C26—C21	119.40 (12)
C17—C12—C11	109.50 (10)	C25—C26—H26A	120.3
C13—C12—C11	128.68 (11)	C21—C26—H26A	120.3
C14—C13—C12	118.21 (11)	N1—C27—H27A	109.5
C14—C13—H13A	120.9	N1—C27—H27B	109.5
C12—C13—H13A	120.9	H27A—C27—H27B	109.5
C13—C14—C15	120.12 (12)	N1—C27—H27C	109.5
C13—C14—H14A	119.9	H27A—C27—H27C	109.5
C15—C14—H14A	119.9	H27B—C27—H27C	109.5
			
O1—C1—C2—C3	16.5 (2)	C9—C10—C11—O3	75.27 (15)
C9—C1—C2—C3	−166.90 (13)	C18—C10—C11—C12	15.76 (12)
O1—C1—C2—C7	−163.84 (13)	C19—C10—C11—C12	144.75 (10)
C9—C1—C2—C7	12.81 (14)	C9—C10—C11—C12	−104.65 (11)
C7—C2—C3—C4	0.6 (2)	O3—C11—C12—C17	171.44 (12)
C1—C2—C3—C4	−179.67 (13)	C10—C11—C12—C17	−8.65 (13)
C2—C3—C4—C5	1.9 (2)	O3—C11—C12—C13	−7.4 (2)
C3—C4—C5—C6	−2.3 (2)	C10—C11—C12—C13	172.53 (12)
C4—C5—C6—C7	0.0 (2)	C17—C12—C13—C14	0.34 (19)
C3—C2—C7—C6	−3.0 (2)	C11—C12—C13—C14	179.03 (12)
C1—C2—C7—C6	177.30 (12)	C12—C13—C14—C15	0.47 (19)
C3—C2—C7—C8	178.12 (12)	C13—C14—C15—C16	−0.9 (2)
C1—C2—C7—C8	−1.62 (14)	C14—C15—C16—C17	0.40 (19)
C5—C6—C7—C2	2.6 (2)	C15—C16—C17—C12	0.41 (18)
C5—C6—C7—C8	−178.72 (13)	C15—C16—C17—C18	−176.36 (12)
C2—C7—C8—O2	167.84 (13)	C13—C12—C17—C16	−0.79 (18)
C6—C7—C8—O2	−11.0 (2)	C11—C12—C17—C16	−179.71 (11)
C2—C7—C8—C9	−10.25 (14)	C13—C12—C17—C18	176.50 (11)
C6—C7—C8—C9	170.92 (13)	C11—C12—C17—C18	−2.42 (14)
C27—N1—C9—C8	−69.28 (14)	C16—C17—C18—C10	−170.69 (12)
C20—N1—C9—C8	156.11 (10)	C12—C17—C18—C10	12.31 (13)
C27—N1—C9—C1	48.61 (15)	C11—C10—C18—C17	−16.54 (12)
C20—N1—C9—C1	−86.00 (13)	C19—C10—C18—C17	−143.39 (10)
C27—N1—C9—C10	168.82 (10)	C9—C10—C18—C17	104.06 (11)
C20—N1—C9—C10	34.20 (12)	C18—C10—C19—C21	48.01 (15)
O2—C8—C9—N1	−34.63 (17)	C11—C10—C19—C21	−74.23 (13)
C7—C8—C9—N1	143.47 (10)	C9—C10—C19—C21	167.49 (10)
O2—C8—C9—C1	−161.28 (12)	C18—C10—C19—C20	−80.86 (12)
C7—C8—C9—C1	16.82 (12)	C11—C10—C19—C20	156.90 (10)
O2—C8—C9—C10	80.66 (15)	C9—C10—C19—C20	38.62 (11)
C7—C8—C9—C10	−101.24 (11)	C9—N1—C20—C19	−9.38 (14)
O1—C1—C9—N1	34.79 (18)	C27—N1—C20—C19	−144.95 (11)
C2—C1—C9—N1	−141.92 (11)	C21—C19—C20—N1	−149.54 (10)
O1—C1—C9—C8	158.94 (12)	C10—C19—C20—N1	−20.02 (13)
C2—C1—C9—C8	−17.76 (12)	C20—C19—C21—C22	−136.87 (12)
O1—C1—C9—C10	−80.86 (15)	C10—C19—C21—C22	100.35 (13)
C2—C1—C9—C10	102.43 (11)	C20—C19—C21—C26	40.61 (16)
N1—C9—C10—C18	79.65 (11)	C10—C19—C21—C26	−82.17 (15)
C8—C9—C10—C18	−42.44 (13)	C26—C21—C22—C23	−0.50 (19)
C1—C9—C10—C18	−155.32 (9)	C19—C21—C22—C23	177.08 (12)
N1—C9—C10—C11	−163.99 (9)	C21—C22—C23—C24	−0.5 (2)
C8—C9—C10—C11	73.92 (12)	C22—C23—C24—C25	1.0 (2)
C1—C9—C10—C11	−38.96 (12)	C23—C24—C25—C26	−0.6 (2)
N1—C9—C10—C19	−44.13 (10)	C23—C24—C25—Br1	179.99 (10)
C8—C9—C10—C19	−166.22 (10)	C24—C25—C26—C21	−0.34 (19)
C1—C9—C10—C19	80.89 (11)	Br1—C25—C26—C21	179.05 (9)
C18—C10—C11—O3	−164.32 (12)	C22—C21—C26—C25	0.89 (18)
C19—C10—C11—O3	−35.33 (17)	C19—C21—C26—C25	−176.60 (11)

### Hydrogen-bond geometry (Å, º)

*Cg*1 is the centroid of the C21–C26 ring.

**Table:**

*D*—H···*A*	*D*—H	H···*A*	*D*···*A*	*D*—H···*A*
C18—H18*B*···O2	0.99	2.39	3.0787 (17)	126
C19—H19*A*···O1	1.00	2.53	3.1649 (17)	121
C4—H4*A*···O3^i^	0.95	2.53	3.4056 (18)	153
C16—H16*A*···N1^ii^	0.95	2.58	3.4912 (16)	161
C20—H20*B*···O1^iii^	0.99	2.44	3.3586 (18)	153
C23—H23*A*···O1^iv^	0.95	2.48	3.3847 (18)	160
C5—H5*A*···*Cg*1^v^	0.95	2.65	3.3386 (17)	130
C15—H15*A*···*Cg*1^vi^	0.95	2.82	3.6623 (15)	149

Symmetry codes: (i) −*x*+1, −*y*+2, −*z*+2; (ii) −*x*, −*y*+1, −*z*+1; (iii) −*x*, −*y*+1, −*z*+2; (iv) −*x*+1, −*y*+1, −*z*+2; (v) *x*, *y*+1, *z*; (vi) −*x*+1, −*y*+1, −*z*+1.

## References

[bb1] Bernstein, J., Davis, R. E., Shimoni, L. & Chang, N.-L. (1995). *Angew. Chem. Int. Ed. Engl.* **34**, 1555–1573.

[bb2] Bruker (2009). *SADABS*, *APEX2* and *SAINT* Bruker AXS Inc., Madison, Wisconsin, USA.

[bb3] Cosier, J. & Glazer, A. M. (1986). *J. Appl. Cryst.* **19**, 105–107.

[bb4] Cremer, D. & Pople, J. A. (1975). *J. Am. Chem. Soc.* **97**, 1354–1358.

[bb5] Sheldrick, G. M. (2008). *Acta Cryst.* A**64**, 112–122. 10.1107/S0108767307043930 18156677

[bb6] Spek, A. L. (2009). *Acta Cryst.* D**65**, 148–155. 10.1107/S090744490804362X PMC2631630 19171970

[bb7] Wei, A. C., Ali, M. A., Choon, T. S., Arshad, S. & Razak, I. A. (2012*a*). *Acta Cryst.* E**68**, o1340–o1341. 10.1107/S1600536812014213 PMC3344476 22590238

[bb8] Wei, A. C., Ali, M. A., Choon, T. S., Razak, I. A. & Arshad, S. (2012*b*). *Acta Cryst.* E**68**, o560–o561. 10.1107/S1600536812003169 PMC3297291 22412481

[bb9] Wei, A. C., Ali, M. A., Yoon, Y. K., Quah, C. K. & Fun, H.-K. (2011). *Acta Cryst.* E**67**, o3274. 10.1107/S1600536811046514 PMC3238931 22199780

